# Analysis of fat, fatty acid profile, and salt content of Iranian restaurant foods during the COVID‐19 pandemic: Strengths, weaknesses, opportunities, and threats analysis

**DOI:** 10.1002/fsn3.2563

**Published:** 2021-09-14

**Authors:** Fatemeh Mohammadi‐Nasrabadi, Azizollaah Zargaraan, Yeganeh Salmani, Abdolsamad Abedi, Ehsan Shoaie, Fatemeh Esfarjani

**Affiliations:** ^1^ National Nutrition and Food Technology Research Institute Faculty of Nutrition Sciences and Food Technology, Food and Nutrition Policy and Planning Research Department Shahid Beheshti University of Medical Sciences Tehran Iran; ^2^ Laboratory of Behshahr Industrial Company Tehran Iran

**Keywords:** fat, fatty acid profile, restaurant foods, salt content, SWOT analysis

## Abstract

This study aimed to analyze the fat, fatty acid profile, and salt content of restaurant foods (RFs) and find out strategies to lower them using the strengths, weaknesses, opportunities, and threats (SWOT). Five types of common foods (*n* = 70) were collected from restaurants in Tehran, Iran. The fat, fatty acid profile, and salt content of samples were analyzed by acid hydrolysis method, gas chromatography, and Charpentier Volhard methods, respectively. The quantitative data were analyzed by the SPSS using ANOVA and Spearman’s correlation test. Then, a SWOT analysis was done. The laboratory results indicated that the highest amount of total fat was in Samosa (16.92% ± 6.27%), whereas saturated fatty acids (SFA) and trans fatty acids (TFA) were significantly higher in Koobideh kebab with rice (44.42% ± 5.07% and 2.86% ± 0.64%, respectively) as compared to other samples. In addition, the highest amount of salt was in the Falafel sandwich (2.87% ± 0.98%). The salt content in the majority of analyzed foods was about two times more than the daily recommendations of the World Health Organization (WHO). The SWOT analysis results showed the lack of standardization of recipes to measure accurate fat and salt content was the main weakness and food labeling in the menus was detected as an important opportunity. TFA and salt in RFs were alarmingly high and it is necessary to find strategies for reformulation of RFs to reduce their fat and salt content. Policymakers can use the SWOT analysis results of this study to offer directions for potential future strengthening actions of healthy foods in restaurants for public health.

## Graphical abstract

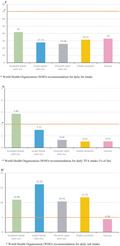

## 1 INTRODUCTION

Nowadays, there are important changes observable in food intake patterns. For instance, there has been a steady increase in the consumption of food away from home because of changing lifestyles and rapid expansion of restaurants (Bahadoran et al., 2012; Statistical Center of Iran, 2016). Consumers spend approximately half of their food budget on meals away from home (Soo et al., 2018). The increase in popularity of ready‐to‐eat meals has been attributed to the scarcity of time to perform household chores (Celnik et al., 2012). Lack of enough time for cooking and the cost‐effectiveness of such products have caused the growth of the ready‐to‐eat meals market (Sgroi et al., 2018).

Restaurant foods are, generally, recognized as a source of fat and salt. An excessive intake of fat, especially saturated and trans fatty acids (SFAs and TFAs) is perceived as the leading cause for an increased risk of noncommunicable diseases (NCDs) development.

Previous studies revealed high consumption of junk foods in the Iranian population, especially in children and adolescents. The most important affecting factors include availability, low price, media advertisements, preference of fast foods taste, the variety of the packages and their attractiveness, low awareness, and changing in lifestyle (Rezazadeh et al., 2017).

In Pakistan, Vanaspati ghee (partially hydrogenated vegetable oil), bakery shortening, hard margarines, and fat spreads are identified as the major sources of TFAs. Federal and provincial food authorities have recently established the limits for TFAs in few products; however, the TFA regulations are insufficient and not in line with global best practices (Rashid et al., 2020).

Linolenic Acid is an essential fatty acid belonging to the omega‐3 fatty acids group. Linoleic acid is a poly‐unsaturated omega‐6 fatty acid. Due to multiple double bonds, poly‐unsaturated fatty acids (principally linolenic acid and to a lesser degree linoleic acid) are also oxidatively unstable. During deep fat frying applications, for example, oxidation at the n‐3 and n‐6 double bonds results in rancid odors and flavors. Partial hydrogenation helps to improve the oxidative stability of soybean oil by chemically reducing the levels of linolenic and linoleic acid. However, this process also generates trans fats, which have been correlated with elevated blood serum LDL cholesterol and increased risk of coronary heart disease (Hunter, 2006).

In addition, high intake of foods rich in salt is related to the increased risk of developing hypertension, which contributes to the burden of several chronic diseases (e.g., obesity, diabetes, cancer, and cardiovascular diseases [CVDs]), stroke, and kidney failure, premature mortality, and disability (World Health Organization [WHO], 2013a).

Healthier meal selections at restaurants are often limited and not actively promoted. On the other hand, fat in RFs is often combined with a salty taste; contributed to higher risk of obesity, CVDs, and cancer (Bolhuis et al., 2018). There is unequivocal evidence that excess sodium intake leads to increased blood pressure, which in turn, results in increased risks of CVDs. While many countries have started to act on reducing sodium intake, further actions and commitments to achieving a 30% reduction in dietary sodium by 2025 are needed (WHO, 2013b). The results of a national study conducted by Iran's National Nutrition & Food Technology Research Institute in 2016 showed that the per capita daily consumption of salt (7 g) and fat (15 g) is more than the recommended amounts (NNFTRI, 2016). There is a clear relationship between habitual sodium intake and blood pressure levels and CVDs. It has been estimated that at least 150,000 premature deaths per year could be averted in the United States if the sodium content of packaged foods and RFs was reduced by 50% (Jacobson et al., 2013). Therefore, for the prevention of CVDs, the WHO recommends a salt intake level of less than 5 g/day for adults and 2 g/day for children (WHO, 2013a).

According to the guidelines, the intake of TFAs should be as low as possible (<1% of total energy intake), which for a 2000 kcal diet, represents around 2 g of TFA intake per day (FAO & WHO, 2008; Leth et al., 2006). Furthermore, based on the rule of the Food and Drug Administration (FDA), the appearance of TFAs in food labeling of all food products in the United States is necessary. As RFs are one of the main sources of TFAs, knowing the amount of this kind of fatty acids along with the other fatty acid contents of these products in Iran seems to be necessary (Chang et al., 2003).

Trans fatty acids intake should be as low as possible within the context of a nutritionally balanced diet (Souza & Lumley, 2015). An excessive intake of TFAs has been linked with an increase in coronary heart disease risk factors and coronary heart disease events (EFSA Panel on Dietetic Products & Allergies, 2010).

Prior to 2007, there was no restriction on the amount of saturated and trans fatty acids in semihydrogenated oils in Iranian policymaking authorities. This year, with the aim of improving the quality of edible oils in the direction of nutritional health of the community, the restriction of saturated and trans fatty acids (maximum amount of 20% and 30%, respectively) in the development of national standards became mandatory. The policy of reducing the amount of trans fatty acids in edible oils has been gradual and this amount decreased to less than 10% in 2011 and to less than 2% in 2015 (Esmaeili et al., 2021).

During the last two decades, many measures have been taken to change the composition of the consumption pattern of oils in order to promote public health in Iran. Fifteen food standards associated with eight food items that make up a large share of the daily Iranian food basket and three that make up a small share were evaluated. Policies on salt included reduction in maximum permitted percentage in bread, cheese, and dough (a fermented drink) to 1%, 3%, and 0.8%, respectively. For trans and saturated fats, maximum permitted percentages were set as 2%–5% and 30%–65% of edible oils and fats, respectively. Nutritional traffic light labeling, which indicates the content of salt, sugar, fat, and trans fat in foods has been mandatory for all foods since 2016 (Moslemi et al., 2020).

A study in 2012 demonstrated that public campaigns and policy measures are motivating restaurants' manager to replace the trans fatty acids in foods with alternative fats in Iran. It was also mentioned that fat intake can be decreased by proper education, voluntary reduction by oil industries and labeling of amount of fat (Peymani et al., 2012). However, despite the great efforts having been made in Iran, there is still room to make the documented policies and their conformities with each other to minimize the amount of TFAs in food products much more efficient (Saghafi et al., 2018).

The WHO has established regulation norms that include the reduction of exposure to foods with an unhealthy amount of fat, SFAs, TFAs, sugar, and salt. It has also recommended continual monitoring to quantify the number of exposures and strategies of the used foods (Hawkes & WHO, 2007). Thus, assessment of salt and fat content of RFs especially trans and saturated fatty acids is necessary and can provide safe food to prevent noncommunicable diseases and improve public health. To our knowledge, this is the first study to evaluate the fat, fatty acid profile, and salt content of RFs during the COVID‐19 pandemic to get an understanding of the current situation and identify the priority intervention. Also, SWOT analysis could offer directions for practical solution of healthy food in restaurants as a priority for public health.

## 2 MATERIALS AND METHODS

### 2.1 Sample selection

This cross‐sectional study was conducted in five districts (North, East, West, South, and Center) of Tehran City (Iran), which were classified as high, moderate, and low socioeconomic status based on the report of the Ministry of Economic and Financial Affairs (Tajali‐Pour & Alikhani, 2012). One hundred restaurants (20 restaurants from each district) were selected randomly through websites from restaurants which served foods takeaway due to the COVID‐19 lockdown. Thirty of them (six from each district) were selected based on popularity. Then, each restaurant's manager was contacted to ask for the bestselling food during the survey. Afterward, five types of bestselling food were identified, including Koobideh kebab (ground lamb or beef with ground pepper, chopped onions, and salt) and rice (with oil, butter, salt, and saffron), Joojeh kebab (broiler, onion, yogurt, bloomed saffron, oil, and salt) and rice, Samosa (fried chopped potato, salt and fried beef sausage in fried bread), Falafel sandwich (chickpeas, vegetable oil and salt with tomatoes, pickled cucumber, and Baguette bread), and Ghormeh sabzi (Persian herb stew: boneless chuck roast, fried vegetables, red kidney beans, and oil) and rice that were widely appreciated by people from all ages. The foods have chosen based on the restaurant union's annually report, and then confirmed with restaurants managers. A total of 70 food samples (14 samples from each food) were ordered blind from the selected restaurants during 1 week in Tehran City, in June 2021, to determine their total fat (%), fatty acid profile (%), and total salt content (%) (Iran National Standards Organization [INSO], 2016, 2018).

### 2.2 Sample preparation

Each day, 10 samples were ordered and encoded. Then samples were transported in a cool box with ice sheets to the quality control laboratory. Upon arrival, an excel form was filled out with the following information: (1) identification; (2) description; (3) place, date, and time of collection; (4) portion size, and (5) list of ingredients.

To obtain 900‐ml homogeneous samples and reducing the errors, the samples were mixed by a blender (GM200, RETSCH). Then they were passed through the paper filter to a dark‐colored PET container (to prevent chemical changes). Next, the samples were filtered based on the food type and stored at −20°C until analyzing. All measurements were repeated two times.

### 2.3 Total fat

Total fat was determined by an acid hydrolysis method (Albuquerque et al., 2016). Approximately 75 ml of ultrapure water and 45 ml of hydrochloric acid fuming 37% were added to 5–10 g of each sample. Then the solution was boiled for 20 min and left to cool down until room temperature. Afterward, the solution was filtered with filter paper (Whatman^®^ no 40). For the fat extraction, a Soxhlet apparatus (Soxtec™ 2050, Auto Fat Extraction System, FOSS Analytical) and petroleum ether were used. The obtained residue was dried for 1 h and 30 min at 101 ± 2°C until achieving constant weight. The analytical procedure for the determination of total fat content was performed in triplicate. The results are expressed as g/100 g for all the analyzed food products.

### 2.4 Fatty acid profiles

Fatty acid methyl esters (FAMEs) were prepared from 0.5 g of oils according to ISO 12,966 (2011). Then they were analyzed using gas chromatography (Agilent 6,890 GC, carrier gas Helium, flame gas H_2_, column HP‐88: 100 m) with a Flame Ionization Detector (FID). The initial oven temperature started at 180°C, hold for 5 min, increased at the rate of 1°C/min to 190°C, hold for 20 min, increased at the rate of 1°C/min to 200°C, and then hold for 17 min. The FID temperature was 220°C, the injection temperature was 210°C, and the retention time was 62 min. The quantification was done by comparing the peak area/height of lipid standards in the sample normalization system (INSO, 2016).

### 2.5 Salt content

The salt content of 100 g foods was determined in duplicate with the Charpentier Volhard method (AOAC, 2016, method number 935.43, SMEDP 15.052) directly on five types of food mixture (AOAC, 2016). Approximately, 3 g of dried and milled food was mixed with 25 ml of 0.1 mol/L silver nitrate and boiled in 15 ml of concentrated nitric acid and 2 ml of potassium permanganate solution. Excess of silver ions was titrated with 0.1 mol/L ammonium thiocyanate in the presence of diethyl ether and ferric ammonium sulfate indicator. Reproducibility was estimated through the calculation of the coefficient of variation for the carried sample analysis (CV% = 4.5). Accuracy was evaluated by calculating the mean percentage recovery of samples spiked with known amounts of sodium chloride at the beginning and the end of each day's work (INSO, 2013). Sodium chloride was estimated from the amount of chloride ion, as determined by the end‐point of titration.

### 2.6 Strengths, weaknesses, opportunities, and threats analysis

A SWOT analysis, as a technique for generating strategic alternatives from situation analysis, is used to assist for identifying a strategic direction for an organization. SWOT stands for strengths, weaknesses, opportunities, and threats. It was preferred for the present work as it yields useful information about the future viability of the considered system. SWOT matrix helps managers or policymakers to develop four types of strategies (invasive strategies [SO], competitive strategies [ST], conservative strategies [WO], and defensive strategies [WT]) and provides a framework for identifying and formulating strategies (Dahlan et al., 2020; Unakitan and Abdikoglu, 2016). SWOT analysis is an instrument to identify strengths, weaknesses, opportunities, and threats to devise an approach to analyze the situation and new strategies (Stolovitch & Keeps, 2006).

Due to the lockdown and social distancing of all citizens during data gathering and to limit the spread of the disease, we preferred to run the study through an online portal. The link of an online question form was sent to the 60 popular restaurant managers, who were selected randomly from the social media, 12 restaurant union members, five nutritionists, and three policymakers by the “WhatsApp” messenger (Facebook, Inc). Out of the 60 restaurant managers and 12 restaurant union members 37 (61%) and 9 (75%) responded, respectively. Totally, 44 (67%) of them answered the question. A question was about their opinions on the strengths, weaknesses, opportunities, and threats (SWOT) of the implementation of the fat and salt reduction program in restaurants.

The results were checked and validated by two stakeholders that met the inclusion criteria but did not participate in the research. For reliability confirmation, two researchers checked the findings again. The results were then shared and summarized to improve accuracy, validity, credibility, and transferability. Then, using the SWOT results, the potentials and limitations of the region were identified and practical solutions were proposed for fat and salt reduction in restaurants.

### 2.7 Statistical analysis

After collecting the required data from the laboratory, the data analysis was performed by the SPSS software (SPSS Inc., version 16). Descriptive statistics, including mean fat, fatty acid, and salt preportion size and 100 g of each RF were presented. Kolmogorov–Smirnov's test was used for checking the normality of the data. Between‐group comparisons were conducted using ANOVA and Kruskal–Wallis tests. In addition, correlations between the variables were tested by Spearman's correlation matrix analyses. The *p*‐values <.05 were considered as statistically significant.

## 3 RESULTS

The results derived from the present study (Table 1) indicated that in 100 g of the tested samples, the highest amount of total fat (16.92 ± 6.27 g) was detected in Samosa, which was significantly higher than in the other RFs, whereas Koobideh kebab with rice had the highest amount of SFAs (44.42% ± 5.07% from fat) and TFA (2.86% ± 0.64% from fat), significantly. The average highest portion size was in Joojehh kebab with rice (630 ± 15.83 g). Also the highest amount of salt in 100 g of the food samples was found in the Falafel sandwich (2.87 ± 0.98 g).

**TABLE 1:** Mean ± SD of portion size, fat, SFA, TFA, and salt content of restaurant foods

Mean ± SD					
Food items (*n* = 70)	Portion size (g)	Fat (%)	Saturated fatty acids (% fat)	Trans fatty acids (% fat)	Salt (%)
Koobideh kebab & rice	450 ± 12.17	9.35 ± 2.37	44.42 ± 5.07^a^	2.86 ± 0.64^b^	2.44 ± 0.19
Falafel sandwich	410 ± 8.21	7.70 ± 5.76^a^	19.20 ± 6.40^b^	0.52 ± 0.22^b^, ^c^	2.87 ± 0.98
Ghormeh Sabzi & rice	480 ± 25.34	5.35 ± 1.34^a^	24.82 ± 3.93^b^	0.66 ± 0.39^b^, ^c^	2.17 ± 0.24
Samosa	195 ± 6.07	16.92 ± 6.27	19.07 ± 2.36^b^	0.53 ± 0.19^b^, ^c^	2.25 ± 0.37
Joojehh kebab & rice	630 ± 15.83	4.40 ± 2.40^a^	41.58 ± 4.78^a^	1.51 ± 0.68^c^	2.57 ± 0.01
Total	134.72 ± 82.75	9.64 ± 6.45	27.00 ± 11.30	1.03 ± 0.93	2.53 ± 0.66

^a^ Significant difference with Samosa by ANOVA (*p* < .05).

^b^ Significant difference with Joojeh kebab & rice by ANOVA (*p* < .05).

^c^ Significant difference with Koobideh kebab & rice by ANOVA (*p* < .05).

Additionally, there was a significant positive association between the total SFA and TFA content of RFs in Iran (r = .858, 95%CI: 0.749–0.936, *p* < 0001); however, no association was found between the salt and fatty acid content of the analyzed foods.

As shown in Table 2, among all unsaturated fatty acids (UFAs), oleic acid (C 18: 1c) and linoleic acid (C 18: 2c) had the highest concentrations in the studied food samples. Palmitic acid (C16: 0), as the most common SFA, was highly observed in Joojehh kebab with rice (27.50 ± 2.26) and Koobideh kebab with rice (24.65 ± 2.76), respectively.

**TABLE 2:** Fatty acid profile of restaurant foods (% of fat)

Restaurant foods (*n* = 70)	Fatty acid profile (% from fat) Mean ± SD												
	Caprylic C8:0	Capric C10:0	Lauric C12:0	Myristic C14:0	Palmitic C16:0	Palmitoleic C16:1	Stearic C18:0	Trans Oleic (C18:1)	Oleic C18:1	Trans Linoleic C18:2	Linoleic C18:2	Linolenic C18:3	Possible source of oils/ fats
Koobide kebab with rice & butter	0.30 ± 0.14	0.7 ± 0.14	1.0 ± 0.00	3.43 ± 1.59	24.65 ± 2.76	2.20 ± 0.62	15.1 ± 2.36	2.56 ± 0.64	37.13 ± 5.57	0.30 ± 0.00	6.23 ± 3.86	0.90 ± 0.62	Sunflower+Animal Fat (from meat)& butter
Falafel	–	–	2.00 ± 0.00	0.36 ± 0.20	15.32 ± 6.36	0.11 ± 0.04	4.12 ± 0.35	0.11 ± 0.13	34.143 ± 4.40	0. 41 ± 0.12	40.83 ± 7.51	2.48 ± 0.60	Soybean & Sunflower
Ghormeh Sabzi with rice	–	–	–	0.35 ± 0.07	14.37 ± 2.22	0.1 ± 0.00	4.35 ± 0.07	0.23 ± 0.04	40.1 ± 0.42	0.3 ± 0.14	35.25 ± 2.05	2.60 ± 0.28	Soybean & Sunflower
Samose	–	–	0.10 ± 0.00	0.44 ± 0.89	19.80 ± 3.73	0.14 ± 0.54	4.52 ± 0.29	0.24 ± 0.24	35.45 ± 4.01	0.42 ± 0.16	34.88 ± 5.25	2.18 ± 0.65	Soybean, Sunflower& Canola
Joojehh kebab with rice& butter	0.53 ± 0.46	0.80 ± 0.42	1.20 ± 0.28	3.05 ± 3.04	27.50 ± 2.26	1.00 ± 0.70	8.50 ± 2.82	1.21 ± 0.68	32.85 ± 2.05	0.30 ± 0.00	19.41 ± 5.38	1.55 ± 0.35	Sunflower+Animal Fat (from meat)& butter
Total	0.41 ± 0.31	0.75 ± 0.26	0.61 ± 0.53	1.24 ± 1.66	19.15 ± 6.25	0.56 ± 0.84	6.44 ± 4.20	0.66 ± 0.95	35.44 ± 4.24	0.37 ± 0.12	30.96 ± 13.76	2.06 ± 0.79	
Possible source of oils/ fats	Animal fat ( meat or butter)	Animal fat ( meat or butter)	Coconut & Palm kernel	< 1% in vegetable oils (Palm, Coconut) & Butter Fat	Almost all vegetable oils	Animal Fat (meat fat)	Almost all vegetable oils	Heat	Corn, Olive, Palm, Soybean, Sunflower	Heat	Sunflower Soybean	Canola Soybean Palm	

**FIGURE 1:**
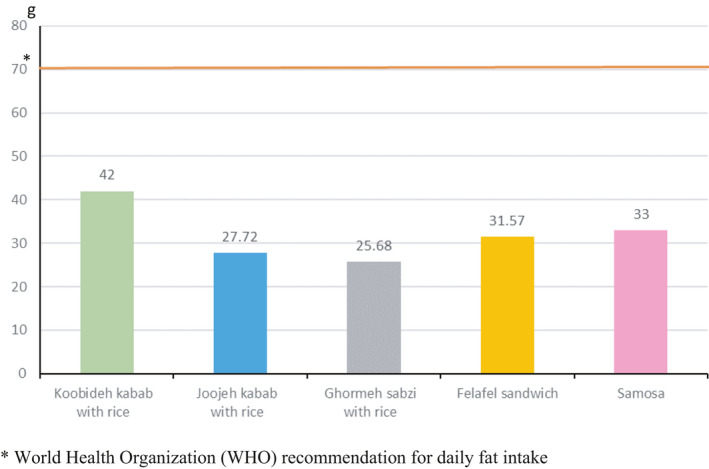
Fat content of restaurant foods per portion (g)

**FIGURE 2:**
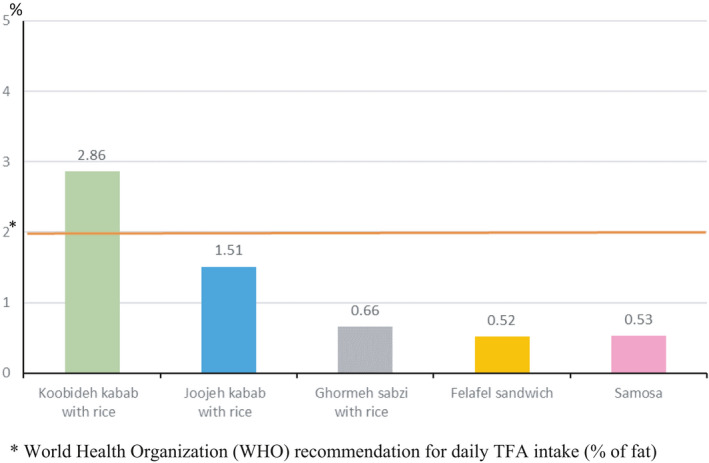
Trans fatty acids of restaurant foods per 100 g of fat. *World Health Organization (WHO) recommendation for daily TFA intake (% of fat)

**FIGURE 3:**
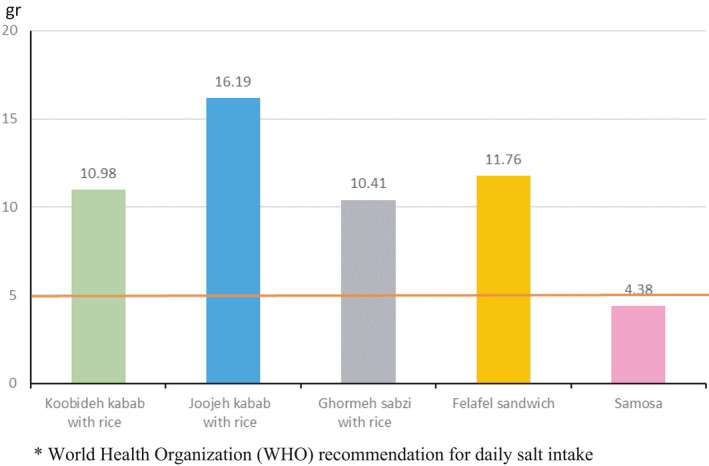
Salt content (g) of restaurant foods per portion. *World Health Organization (WHO) recommendation for daily salt intake

The fat content per portion of RFs samples is displayed in Figure 1. The amount of fat content in Koobideh kebab with rice was the highest per portion. Overall, the results indicated that all food samples had about 50% of the daily WHO recommended fat. However, the amount of TFA in a portion of Koobideh kebab with rice was more than the WHO recommendation for daily TFA intake (Figure 2). In addition, the highest amount of salt‐based on portion size was found in Joojehh kebab with rice (Figure 3).

The final results of the SWOT analysis identified two strengths, eight weaknesses, thirteen opportunities, and four threats. Also, analysis showed the lack of standardization of recipes to measure accurate fat and salt content was the main weakness and food labeling in the menus was detected as an important opportunity. On the other hand, the lack of social and political attention and support was the one of the most important threats (Table 3).

**TABLE 3:** Strengths, weaknesses, opportunities, and threats matrix of fat and salt reduction in restaurant foods

Internal factors	
Strength	Increasing awareness by nutritionists Consumer demand for low‐fat and low‐salt foods for patients in restaurant menu
Weakness	Lack of standardization of recipes to measure accurate fat and salt content Lack of enough food quality control in restaurants Traditional cooking style instead of using modern technology for cooking Mismanagement for healthy cooking Poor innovation and limited technical expertise to design menu for low‐fat and low‐salt foods Lack of governance structures to coordinate, manage and control restaurant foods Lack of training for the food service staff on healthy culinary techniques Lack of knowledge in consumers, especially with low socioeconomic status

External factors	
Opportunity	Establishment of food labeling in menus Reducing saturated fats to less than 10% of total energy intake Reducing trans fats to less than 1% of total energy intake Increasing free specialized and technical training programs for managers and food handlers Consumer awareness campaigns Supply of raw materials with low fat and low salt in food industry Replacing saturated fats with unsaturated, mono‐ and poly‐unsaturated fats Limiting the amount of salt and high‐sodium condiments when cooking and preparing foods Not having salt shaker or high‐sodium sauces on the tables Modifying the portion sizes Implementing the standardization of recipes to measure accurate fat and salt content Developing healthy incentive programs by the restaurants Reformulation of recipes to reduce the fat and salt content
Threat	Lack of social and political attention and support Limited control on food advertisements High price of healthy foods in restaurants Increasing the spread of noncommunicable diseases and increasing the cost of treatment

All the data were analyzed to find the internal and external factors to be used as a basis for decision‐making strategies to reduce fat and salt content in RFs. The most important strategies include as follows: Reformulation of recipes to reduce the fat and salt content (Invasive strategies), Establishment of food labeling in menus (Competitive strategies), Healthy cooking training in the restaurants (Conservative strategies) Controlling price of the healthy foods in restaurants (Defensive strategies; Table 4).

**TABLE 4:** Strategies for fat and salt reduction in restaurant foods

Invasive strategies (SO)	Conservative strategies (WO)
Implementation of traceability in restaurants Increasing political attention to this issue Increasing public awareness by campaign Reformulation of recipes to reduce the fat and salt content	Setting new regulation for restaurants Implementation of food quality control in restaurants Innovation in design menu for low‐fat and low‐salt foods Healthy cooking training in the restaurants
Competitive strategies (ST) Implementation specific licensing system in place for health‐oriented menus in restaurants More interaction between governmental sectors and restaurants' union for increasing public health Using modern equipment in restaurants Establishment of food labeling in menus	Defensive strategies (WT) Using modern methods for cooking Controlling food advertisements Controlling price of the healthy foods in restaurants

## 4 DISCUSSION

Based on the research results, the highest amount of total fat was detected in Samosa, and Koobideh kebab with rice and butter had the highest amount of TFA. Also the salt content in Falafel sandwich was the highest.

The findings of a study about food consumption patterns and the intake of fat and salt in Indonesia (2021) showed that the major food source contributing to the salt and fat intake included RFs (Andarwulan et al., 2021). To avoid unhealthy weight gain, total fat intake should not exceed 30% of total energy intake (FAO & WHO, 2008; Hooper et al., 2015). Intake of SFAs should be less than 10% of total energy intake, and intake of TFAs should be less than 1% of total energy intake, with a shift in fat consumption away from saturated fats and TFAs to unsaturated fats, and in line to eliminate industrially produced TFAs (FAO & WHO, 2008; Nishida & Uauy, 2009).

The dietary reference intake for fat is 70 g/day for adults (Regulation (EU) No (1169)/2011, 2011), but a study in Iran showed that the per capita consumption of fat is more than the WHO recommended amounts (NNFTRI, 2016). However, our findings imply that the average fat intake in one portion of the RFs was approximately in the range of 16.7–42 g. While consuming other foods during the day might increase the intake more than the WHO recommendation. Also, the analysis of dietary intake has revealed that Iranians consume twice as much TFAs as the US population (Mozaffarian et al., 2007).

Trans fatty acids is the common name for a type of unsaturated fat with at least one double bond in the trans configuration that is not synthesized by the human body and is not required in the diet. An excessive intake of TFAs can increase the risk of noncommunicable diseases (Mozaffarian et al., 2006). Frying oils and hydrogenated oils are the major sources of TFAs (Azadbakht & Esmaillzadeh, 2012). In fried foods, trans unsaturations start to increase at 150°C and become much more significant beyond 250°C. After heating for 20 min at 200, 250, and 300°C, there is an increase of 356.5%, 773.9%, and 3026.1%, respectively, in the concentration of Trans isomers to the initial values (0.22 mg/g) (Moreno et al., 1999).

Based on different levels of fats and oils used in manufacturing, the TFAs content of fast foods varies greatly between different countries (<1 g/serving to 24 g/serving) and even within countries (<1 g/serving to 5 g/serving) (Kirkpatrick et al., 2006). According to the WHO recommendations, TFA intake should be as low as possible within the context of a nutritionally balanced diet (EFSA Panel on Dietetic Products & Allergies, 2010; European Food Safety Authority (EFSA), 2017).

In Iran, the content of TFAs in RFs can be reduced by decreasing the amount of partial hydrogenated fat usage during the preparation of RFs (Asgary et al., 2009). TFA consumption in Iran could be reduced by recognition and avoidance of using partially hydrogenated oils by consumers, voluntary reductions in their use by restaurants and food manufacturers, or governmental intervention to limit their usage. In Iran, each individual is entitled to an annual ration of inexpensive (i.e., heavily subsidized) cooking oil, currently fulfilled by the sales of partially hydrogenated oils. Governmental legislation to subsidize unhydrogenated rather than partially hydrogenated oils would be a good strategy in this regard (Mozaffarian et al., 2007).

Results of Esmaeili et al., showed that quantity of the Trans fatty acids in partially hydrogenated vegetable oils has decreased by 95%, compared to that in previous decade. Intakes of Trans fatty acids ranged from 0.32 to 0.67 g/person/day. Moreover, consumption of partially hydrogenated vegetable oils by households has decreased by 45%, compared to that in the last decade (Esmaeili et al., 2021).

Reducing the intake of partially hydrogenated oils in Iran through governmental intervention is feasible and would likely result in substantial health benefits. The incidence of CHD in Iran has been increasing is likely to continue unless other underlying causes are also addressed, including smoking, overweight, low physical activity, and other unhealthy dietary habits in addition to the intake of partially hydrogenated oils.

According to the European Strategy for Prevention and Control of Noncommunicable Diseases 2012–2016, the “elimination of trans fats in food (and their replacement with poly‐unsaturated fats)” is necessary (WHO, 2012, 2016). This is also a part of the European Food and Nutrition Action Plan 2015–2020, that general objective is to improve food system governance, increase the overall quality of the public nutritional status, and to promote health and well‐being (WHO, 2014).

The highest percentage of SFAs was observed in Koobideh kebab, which due is to the presence of meat fat in these kebabs. Another study in Iran reported the same results (Pasdar et al., 2013).

Among all UFAs, oleic acid and linoleic acid had the highest concentrations in the food samples of this study. A study in Iran demonstrated that, the highest percentage in the product containing canola oil is related to oleic acid and in the product containing soybean oil is related to linoleic acid (Moslemy et al., 2010). So, it seems that in this study major source of the edible oils were used for cooking of food samples consist of canola and soybean sunflower oils. Also, using butter in two food samples (Koobideh kebab with rice and Joojeh kebab with rice), was a source of short‐chain fatty acids (Caprylic, Capric) in this analysis.

Among the USFAs, palmitic acid was the main fatty acid in the RFs in this study. Studies show that palmitic acid (PA), a common fatty acid in the human diet, serves as a signaling molecule regulating the progression and reduction of many diseases at the molecular level. The focus of PA regulatory roles is in the reduction of metabolic syndrome, CVDs, cancer, neurodegenerative diseases, and inflammation (Fatima et al., 2019). On the other hand, a study in Iran showed that, the combination of linoleic and linolenic acid may improve the indexes of palmitic acid (Hashem Zadeh et al., 2018).

Another nutrient, which its intake in many countries is far above the recommended level and is different from country to country, is salt. In most European countries, the daily salt intake ranges from 7–12 g. Most of these countries have recommended 5 g per day (Rasmussen et al., 2010). Keeping daily salt intake to less than 5 g (equivalent to the daily sodium intake of less than 2 g) helps to prevent hypertension and reduce the risk of heart diseases and stroke in the adult population (WHO, 2012, 2016). However, in many cases, they are consuming much more than the current WHO recommendation such that it has turned into a public health issue (Paul & Brown, 2007).

Sodium is an essential constituent of salt. Hence, too much consumption of it can contribute to high blood pressure (Rekha et al., 2013). Eighty percent of the sodium consumed by Americans and 77% of dietary sodium intake Canadians are added by processed food and RFs (Garriguet, 2007; Jacobson et al., 2013). Therefore, considering the prevalence of eating outside the home, the pervasiveness of hypertension and its associated health risks are increased (Canadian Hypertension Education Program, 2006; Garriguet, 2007).

The salt content of the restaurant foods in this study exceeds (twice) the WHO recommendations for most of the foods, representing a high risk for public health. It is worth noting that the salt added at the table has not been included in the present study and maybe it is consumed more than shown.

Bread is one of the staple foods in many cultures and societies including Iran. In a study carried out by Hadian et al, 151 samples including traditional and industrial breads were collected randomly from various bakeries and markets of Tehran. Salt content (sodium chloride) in traditional (Sangak, Barbari, Taftoun, and Lavash) and industrial (Volume and semivolume) breads was investigated according to Volhard method of National Standard. Nearly 93%, 21%, 38%, and 43% of the salt content of Sangak, Barbari, Lavash, and Taftoon, respectively, included in the national salt limit were found to be compliant (Hadian et al., 2020).

Another study assessed the quantities of salt, total fat, saturated fatty acids, and trans fatty acids in 69 samples, including 18 samples of industrial (potato chips, shoestring potatoes, fried onions and fried garlics) and 51 guild (potato chips, string potatoes, fried onions, and fried garlics) fried products from various regions of Iran and their conformities with national standards. The total trans fatty acids in oily phase of all samples was at maximum level, specified for industrial and guild frying oils by the national standard; whereas salt of 18% of industrial potato chips was higher than national standards (Khoshtinat et al., 2019).

Accordingly, it seems that reducing sodium intake should be one of the most important goals of global and national programs for decreasing noncommunicable diseases and is an urgent public health need.

Moreover, the results of this study represent the high fat and salt content only in the main dish and do not include the entire meal, such as salads, soups, and side dishes like fried potatoes, beverages, and dessert, which may be consumed alongside and would further increase the amount of fat and salt consumed when eating out. Another study in Iran demonstrated that, the majority of oil from fast food restaurants were over degraded containing hazardous secondary oxidative products, too (Esfarjani et al., 2019).

### 4.1 Practical solutions

The SWOT analysis of this study discovered the specific challenges and provided the strategic program in order to reduce the intakes of fat and salt. These data can be used by policymakers to set planning, make strategies, and guide target set for this sector. They can also use the results for setting laws and regulations for the restaurant industry to make healthy choices, which is a priority for public health (Table 4).

Another study in Iran suggested to use novel protocols for decreasing oil absorption and to revise the relevant national standards. Also it is mentioned that high levels of total fat and hence high intakes of saturated fatty acids in the products urge necessary revisions of the national standards (Khoshtinat et al., 2019).

Our data suggested that eating out regularly may be harmful, particularly among the large proportion of at‐risk adults. On the other hand, reduction strategies cannot exclusively rely on decreasing portion sizes, but must also emphasize decreases in fat and salt density to decrease their levels per portion. In addition, a comprehensive timely assessment of current fat and salt levels of RFs in other provinces and cities is necessary in order to create reformulation strategies and monitor the progress in this regard.

Furthermore, future longitudinal studies to assess fat and salt reduction progress are needed. Also future studies should be done to determine the amount of total dietary intakes that come from Rfs, as well as the total intake of TFAs and determine their possible effects on human health. Policymakers can make use of the above recommended strategies and solution for setting laws and regulations to increase public health.

### 4.2 Limitations

We should consider some limitations. First, due to the COVID‐19 pandemic and lockdown, some of the restaurants were closed. The amount of salt and fat, which was measured in the food samples, does not represent their intake, because the amount of consumption varies from person to person (Someone may consume more than or less than one portion). On the other hand, people may add salt or butter to the food according to their taste.

Therefore, due to lockdown and budget limitations, our samples may not be reprehensive of all restaurants in Iran. Moreover, this study represents the fat and salt content in foods available in restaurants and does not necessarily reflect consumption.

## 5 CONCLUSION

The research findings revealed that the level of TFA and salt in RFs is alarmingly high so it is necessary to find strategies for the reformulation of the RFs to reduce fat and salt content. This could allow consumers in particular to make more informed decisions and leading consumers to believe that the meals offered are balanced when they are not. It is recommended to develop and establish the menu labeling regulations for restaurants in order to clearly inform their customers about the fat and salt content of the menu options. Policymakers can make use of the practical solutions in this study to offer directions for potential future strengthening actions of healthy food in restaurants as a priority for public health.

## CONFLICT OF INTEREST

The authors declare that they have no conflicts of interest to disclose. This study received no specific grant from any funding sources (commercial or nonprofit sectors).

## ETHICAL APPROVAL

This study was conducted according to the guidelines laid down in the Declaration of Helsinki, and all procedures involving the research study participants were approved by the Research Council of National Nutrition and Food Technology Research Institute. Written informed consent was obtained from all stockholders. Ethical issues (including plagiarism, informed consent, misconduct, data fabrication and/or falsification, double publication, and/or submission, redundancy, etc.) have been completely observed by the authors.
